# Toward a Multidimensional Understanding of Later Life Disability: A Latent Profile Analysis

**DOI:** 10.1093/geroni/igab046.778

**Published:** 2021-12-17

**Authors:** Natasha Peterson, Jeongeun Lee, Eva Kahana

**Affiliations:** 1 Iowa State University, Iowa State University, Iowa, United States; 2 Iowa State University, Ames, Iowa, United States; 3 Case Western Reserve University, Cleveland, Ohio, United States

## Abstract

Disability is difficult to define succinctly. Current literature on disability has primarily focused on physical functional limitations. However, relying on a single dimension or index cannot accurately represent disability as the experience of disability is nuanced and complex. To address these gaps, this study aims to understand the multidimensional nature of disability among retired, community-dwelling older adults. Using a sample of 414 older adults between the ages of 72 and 106 years (M=84.84, SD=4.56), latent profile analysis was employed to identify classes based on five indicators of disability across three domains. The five indicators of disability included difficulties with activities of daily living (ADLs), cognitive impairment, physical impairment, sensory impairment, and participation restrictions. Three classes were found to represent the data best. The most favorable and highly functioning group comprised the highest number of participants (n=242, 59.5%). The next group, class 2 (n=157, 37.9%), was characterized by high physical impairment and ADL-difficulty. The smallest group, class 3 (n=15, 3.6%), had the highest ADL-difficulty and participation restrictions but drastically lower cognitive and sensory impairment. Multinomial logistic regression revealed that class membership was related to sociodemographic characteristics. Finally, class membership predicted several mental health outcomes such as depressive symptoms, positive affect, and life satisfaction in the expected direction. If supported by future work, these findings could inform practitioners in developing more specific interventions relevant to older adults based on their disability profiles. Understanding various combinations of disablement has potential implications for services and interventions to be tailored to individuals’ distinct disability-related needs.

